# Nonmetallic tension band fixation is a viable and low-complication surgical technique in patellar fractures: a five-year retrospective study

**DOI:** 10.1007/s00590-024-03887-w

**Published:** 2024-03-26

**Authors:** Rovere Giuseppe, Romeo Michele, Farinelli Luca, Giancani Michele, Gangi Giuseppe, Manuri Valentina, Fortunato Giustra, Francesco Bosco, Lawrence Camarda

**Affiliations:** 1 Department of Clinical Science and Translational Medicine, Section of Orthopaedics and Traumatology, University of Rome “Tor Vergata”, Rome, Italy; 2 Department of Orthopedic and Traumatology (DICHIRONS), University of Palermo, Via del Vespro, 129, 90127 Palermo, Italy; 3 Clinica Ortopedica Dell’Adulto E Pediatrica Dipartimento Di Scienze Cliniche E Molecolari, Università Politecnica Delle Marche, Ancona, Italy; 4 Department of Orthopaedics and Traumatology, Ospedale San Giovanni Bosco—ASL Città di Torino, Turin, Italy; 5 Department of Orthopaedics and Traumatology, G.F. Ingrassia Hospital Unit, ASP 6, Palermo, Italy; 6 Department of Precision Medicine in Medical, Surgical and Critical Care (Me.Pre.C.C.), University of Palermo, Palermo, Italy

**Keywords:** Patellar fracture, Nonmetallic fixation, Knee, Suture tension band

## Abstract

**Background:**

Traditionally, patellar fractures (PFs) have been managed using metallic tension band fixation, a method often associated with a notable rate of complications. Considering these challenges, this study explores the potential of nonmetallic fixation as a treatment option for PFs. This research aims to provide robust evidence supporting the use of the nonmetallic tension band fixation technique as an effective alternative to conventional metallic tension band fixation, thereby advancing the standard of care in treating these fractures.

**Methods:**

This retrospective study analyzed a consecutive patient series presenting with PFs from 2008 to 2021, treated with a nonmetallic tension band fixation technique. Inclusion criteria were strictly defined to include individuals over 18 years of age with isolated PFs requiring surgical intervention. The study focused on evaluating postoperative complications and clinical outcomes, as measured by standardized scoring systems, at the final follow-up point to assess the efficacy and safety of the employed surgical technique.

**Results:**

In this study, with a mean follow-up of 64 ± 7 months, a total of 64 patients who received open reduction and internal fixation (ORIF) for PFs were enrolled. Among these, five cases required additional surgical interventions. Specifically, two cases were due to knee stiffness, while the remaining three involved complications such as superficial infection, skin irritation, or delayed wound healing. The mean postoperative values recorded for the Western Ontario and McMaster Universities Osteoarthritis Index (WOMAC) score, Oxford knee score (OKS), and visual analog scale (VAS) were 20.4 ± 2.3, 35.5 ± 5.3, and 1.6 ± 0.4, respectively. There were no complications related to the nonmetallic fixation technique or instances of loss of reduction.

**Conclusion:**

This study substantiates that nonmetallic tension band fixation is a safe and effective alternative to traditional metallic tension band fixation for patellar fractures. The study's low-complication rate and reoperation frequency underscore the value of nonmetallic implants in mitigating adverse effects and enhancing clinical outcomes.

*Level of evidence:* IV.

## Introduction

Patellar fractures (PFs), which account for about one percent of all skeletal fractures, are generally caused by low-energy trauma and are more common in women older than 65 years [1–3]. Until the nineteenth century, PFs were treated with splints in extension [4]; since around the 1950s, Muller et al. introduced the anterior tension band technique for the surgical treatment of these fractures [5]. Since then, several techniques, including tension wire constructions, sutures/anchors, screws, and hybrid techniques, have been developed; however, the metal tension band is still the standard approach in the surgical treatment of PFs [6, 7]. Metal implants, however, have been associated with several complications requiring subsequent implant removal; common ones include limitation of range of motion, irritation, or infection of overlying tissues [8]. Solutions such as non-resorbable ultra-high molecular weight polyethylene (UHMWPE) sutures have been applied as an alternative to metal tension bands to reduce these complications [9, 10]. A recent systematic review and meta-analysis [11] comparing clinical outcomes and reintervention rates of metallic and nonmetallic implants reported that both techniques resulted in similar functional and final range of motion outcomes. However, metallic implants were associated with subsequent implant removal [11]. Camarda et al. [12], in their systematic review of the literature, reported an intervention rate for the removal of nonmetallic implants in 3.2 percent of patients. Lozaro et al. [13], in their study, reported interventions for metallic implant removal in 37 percent of patients. A recent cadaveric biomechanical study on 19 simple transverse PFs compared the displacement of the synthesized fracture with classical metal tension cerclage and suture tension bands. No differences in fracture breakdown were reported between the two systems analyzed, demonstrating how nonmetallic implants are a safe and effective alternative [14]. This study analyzes patient-reported outcome measures (PROMs) and complications of patients undergoing nonmetallic fixation treatment for PFs with a mean follow-up of 5 years. The aim is to examine whether this technique could be safely used instead of classic metal tension, reporting similar clinical outcomes but lower complication rates.

## Materials and methods

### Study design

This retrospective study focused on a consecutive series of patients who underwent surgical treatment for PFs at the Orthopaedic and Traumatology Unit of AOUP "Paolo Giaccone" in Palermo, Italy, from January 2008 to June 2021.

### Inclusion and exclusion criteria

The study's inclusion criteria targeted patients who sustained PFs and subsequently received open reduction and internal fixation (ORIF) employing a nonmetallic fixation technique. The specific inclusion parameters involved an age range of 18 to 65 years and a follow-up period of at least 24-month post-surgery. Surgical eligibility was contingent upon specific clinical findings: extensor apparatus insufficiency and the degree of fracture displacement. Fracture displacement was quantitatively defined by two key measurements: an interfragmentary gap ranging from 1 to 4 mm and a cartilaginous step-off of more than 2 to 3 mm. The exclusion criteria included the presence of open fractures, a history of prior knee surgery, instances of polytrauma, and any medical condition that might significantly impact the patient's ability to undergo physical rehabilitation. This encompassed previous difficulties in walking, ipsilateral fractures, severe neurological health disorders, advanced respiratory or cardiological conditions, severe obesity, and chronic pain syndromes.

### Surgical technique

**Fig. 1:**
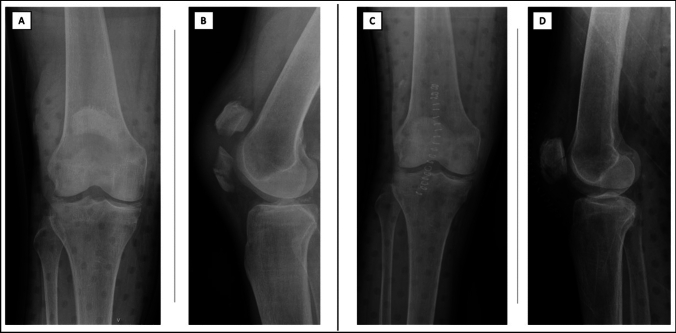
Pre- and postoperative imaging of patellar fractures treated with nonmetallic open reduction and internal fixation (ORIF). **A** preoperative anteroposterior X-ray and **B** preoperative lateral X-ray.** C** postoperative anteroposterior X-ray and **D** postoperative lateral X-ray

**Fig. 2:**
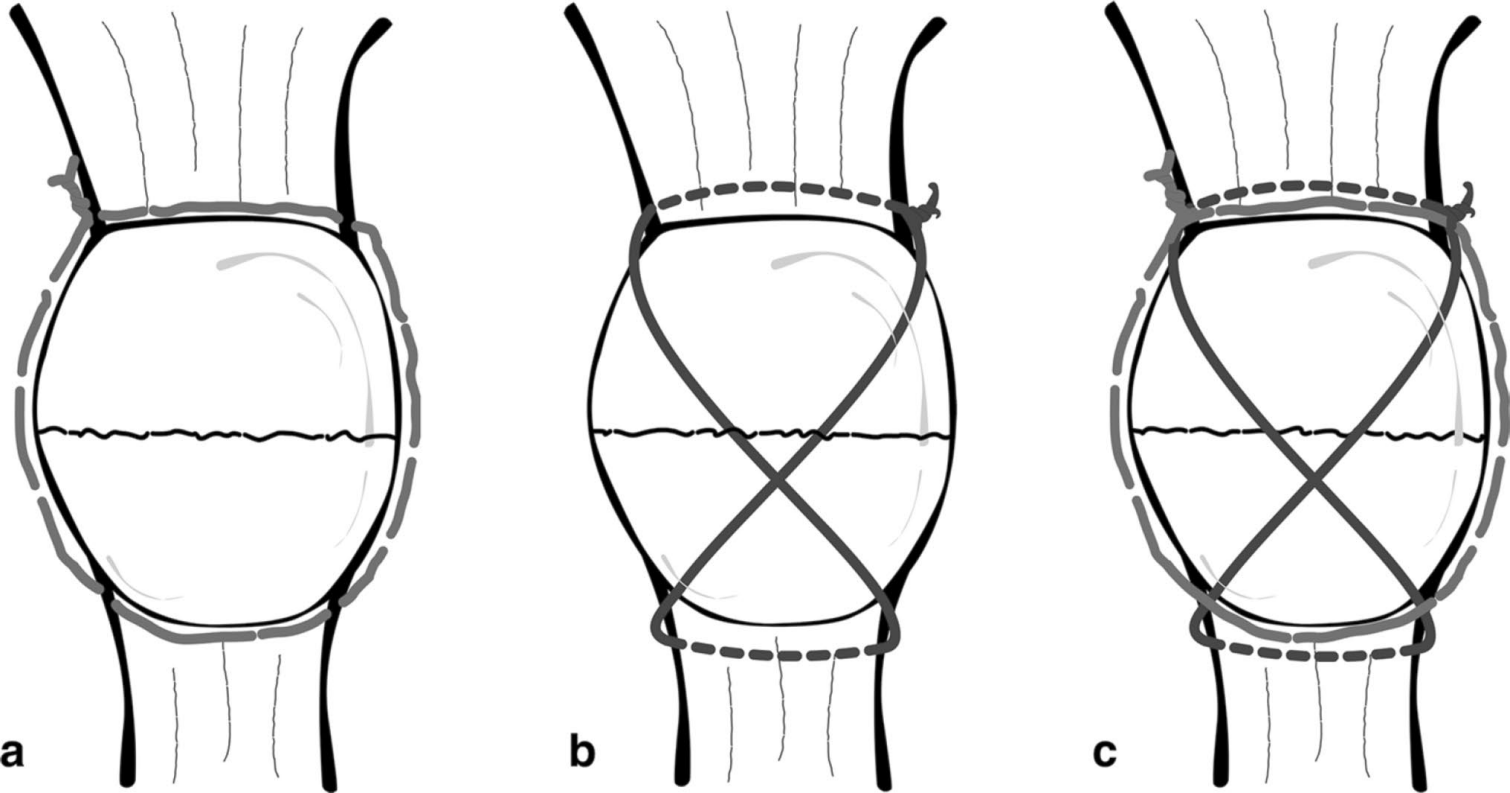
FiberWire suture tension band. **a** Peripatellar circumferential cerclage; **b** modified anterior tension band; **c** final construct. The source is published under a creative commons license from Camarda et al. [9]

Preoperative radiographic planning was conducted using anteroposterior and lateral knee X-ray views (Fig. 1). The surgical procedure was performed by an experienced knee surgeon (LC). The patient was positioned supine, and a tourniquet was applied to the affected thigh. Preoperative fluoroscopy was utilized to guide the surgery. The procedure began with an anterior midline skin incision, followed by a reduction of the articular surface using a reduction clamp, with verification under fluoroscopic guidance. A modified Pyriford technique was employed, utilizing two No. 5 FiberWire® sutures [9]. The first No. 5 FiberWire® suture was used to construct a peripatellar circumferential cerclage, secured and tensioned with five knots, providing preliminary stabilization of the patellar fracture. A second No. 5 FiberWire® suture was threaded through the quadriceps and patellar tendons, arranged in a figure-of-eight configuration to create a modified anterior tension band. During the surgical procedure, if a retinaculum defect was detected intraoperatively, it was addressed with additional repair using nonabsorbable sutures (No. 2 FiberWire®). The anatomical reduction was visually and palpably confirmed, with a step-off of 2 mm considered acceptable. Subsequently, postoperative anteroposterior and lateral knee X-ray views were acquired to validate the reduction's adequacy and assess the synthesis's stability Fig. 1. Further insights into the surgical procedure are illustrated in Fig. 2.

### Postoperative protocol

After surgery, all patients received postoperative care involving immobilization with a long-hinged knee brace, which was locked in the extended position for the first three weeks. Passive range of motion (ROM) exercises began in the third week postoperatively, initially restricted to 090°, focusing on active flexion and extension exercises. Full ROM exercises were permitted starting from the sixth week postoperatively. Patients were allowed gradual weight-bearing as tolerated with the assistance of crutches, progressing to full weight-bearing from the fourth week post-surgery. Regular radiographic monitoring was an integral part of the postoperative protocol, with anteroposterior and lateral knee X-ray views obtained at four-week intervals during the first 6 months after surgery and at the final follow-up visit to assess bony union. Clinical evaluations throughout the follow-up period included assessments using the Western Ontario and McMaster Universities Osteoarthritis (WOMAC) score, the oxford knee score (OKS), and the visual analog scale (VAS). These evaluations were critical for documenting whether subsequent surgical interventions, such as implant removal or additional knee surgeries, were necessary.

### Data extraction

A comprehensive set of demographic data was recorded for each patient using a standardized template. This included details of enrolled patients, types of treatments (conservative and surgical), duration of follow-up, gender, age at the time of surgery, and the affected knee's side. During the follow-up period, clinical and functional outcomes were assessed for all patients using the WOMAC score, the OKS, and the VAS. Data on postoperative complications such as knee stiffness, superficial infections, skin irritation, and delayed wound healing were recorded and analyzed. Each patient underwent a thorough evaluation, including the acquisition of radiographic images. These images were classified using the Orthopaedic Trauma Association's comprehensive fracture classification system (AO/OTA). Four authors (MR, MG, GG, and VM) conducted the data collection process using a standardized proforma. This investigation involved direct patient examinations, reviews of clinical evaluation tables, and telephone interviews. In cases of discrepancies or uncertainties, an experienced knee surgeon (LC) was consulted to ensure the accuracy of the collected data and provide resolution.

### Ethical approval

This study was categorized as exempt from Institutional Review Board (IRB) approval by the IRB of the author's institution. The exemption was granted because it was a retrospective study on a well-established surgical procedure. Despite the exemption, the study was meticulously conducted in alignment with the ethical principles outlined in the 1964 Helsinki Declaration and its subsequent amendments.

### Statistical analysis

Descriptive statistical analysis was performed on the patient cohort data utilizing IBM SPSS Statistics software (version 25.0). This analysis involved calculating mean values for continuous variables, accompanied by their standard deviation (SD), to quantify variability. In the case of categorical variables, computations were made for the absolute numbers and their respective frequency distributions, providing a comprehensive statistical overview.

## Results

### Study cohort and demographics characteristics

**Fig. 3:**
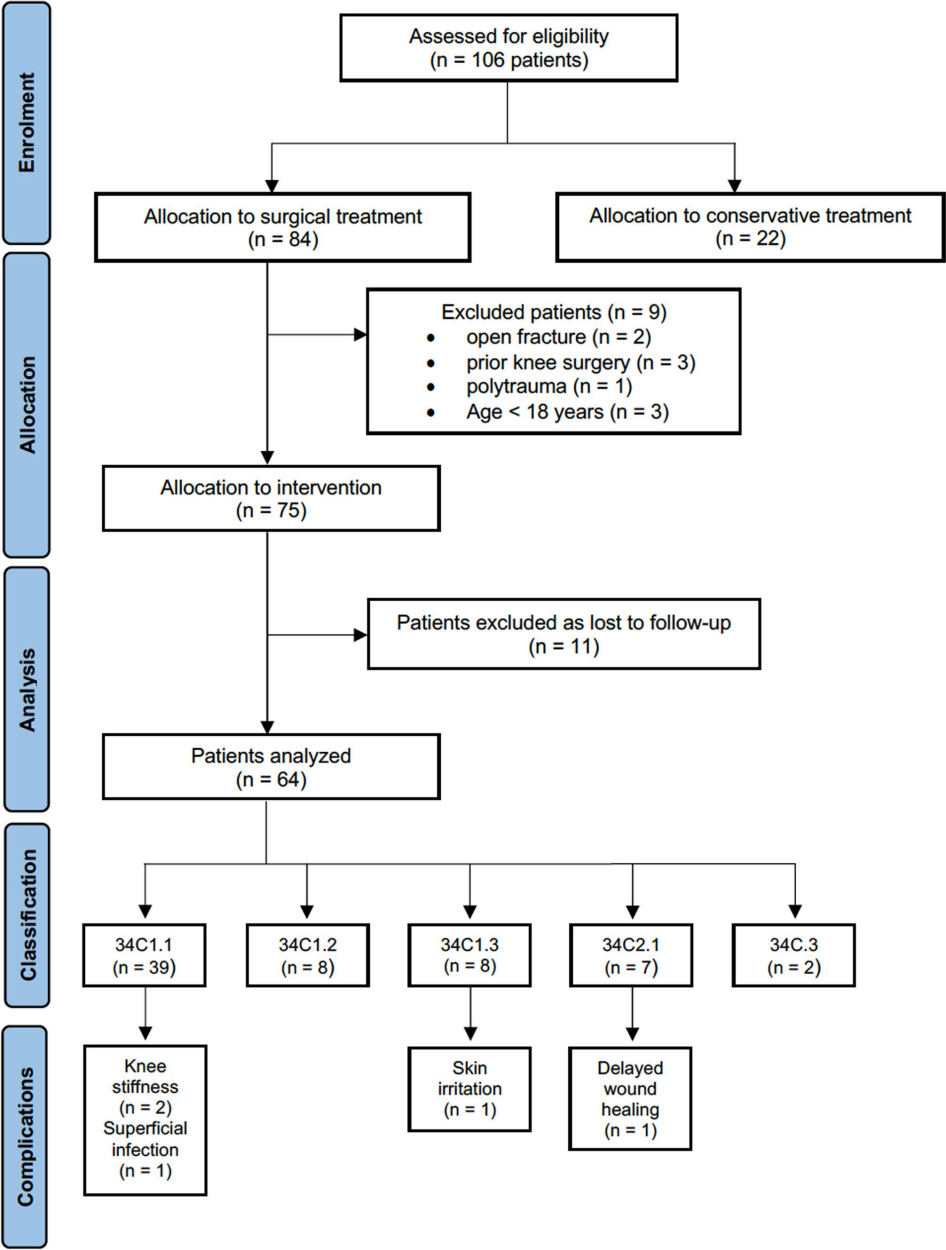
Flow chart of patient enrollment in the study: comprehensive overview. N, number of evaluation cases

From January 2008 to June 2021, our study evaluated a total of 106 patients with patellar fractures (PFs). Among them, 22 received conservative treatment, while 84 underwent surgical treatment involving open reduction and internal fixation (ORIF). However, nine patients were excluded due to non-compliance with the inclusion criteria. Additionally, 11 patients were lost to follow-up, resulting in a final cohort of 64 patients for the follow-up assessment, with a mean follow-up duration of 64 ± 7 months. A flow chart of patient enrollment in the study is shown in Fig. 3. The composition of the final cohort of patients consisted of 38 females and 26 males, with a mean age at the time of injury of 55 ± 6.9 years. The average surgical time was 48 ± 8 min. Fracture distribution was slightly more common on the left side, with 35 patients experiencing left-sided fractures and 29 with right-sided fractures. Detailed demographic data are presented in Table 1.

**Table 1:** Demographic data of patients with patellar fractures treated with nonmetallic open reduction and internal fixation (ORIF)

Demographic characteristics of patients collected					
Total patients	Age at the time of surgery (y.o.)	Sex	Side affected	Surgical time (minutes)	Follow-up (months)
N°	Mean ± SD	M (%)	Right (%)	Mean ± SD	Mean ± SD
64	55 ± 6.9	26 (40.6)	29 (45.3)	48 ± 8	64 ± 7

N°, number of evaluation cases; y.o., years old; SD, standard deviation; M, male; %, percentage

### Type of fracture and postoperative complications

The types of fracture according to the AO/OTA classification were as follows: 39 cases of type 34C1.1, 8 of type 34C1.2, 8 of type 34C1.3, 7 of type 34C2.1, and 2 of type 34C.3 Fig. 3. An analysis of postoperative complications revealed that five patients required subsequent surgical interventions. Specifically, two patients underwent release under anesthesia for knee stiffness, defined as a range of motion (ROM) with maximum flexion of 60°, 3-month post-surgery, following at least 20 physiotherapy sessions. In comparison, three patients required surgeries for superficial infection, skin irritation, and delayed wound healing (one case each) Fig. 3. These complications manifested 6-month post-ORIF.

### Clinical outcomes evaluation

Clinically, at the final follow-up, the mean WOMAC score was 20.4 ± 2.3, the mean OKS was 35.5 ± 5.3, and the mean VAS was 1.6 ± 0.4. Notably, no cases of healing defects were observed. Comprehensive data on postoperative clinical evaluations are detailed in Table 2.

**Table 2:** Postoperative clinical assessment

Postoperative patient-reported outcome measures (PROMs)		
WOMAC	OKS	VAS
Mean ± SD	Mean ± SD	Mean ± SD
20.4 ± 2.3	35.5 ± 5.3	1.6 ± 0.4

SD, standard deviation; WOMAC, Western Ontario and McMaster Universities Osteoarthritis; OKS, Oxford knee score; VAS, visual analog scale

## Discussion

The main finding of this study was to demonstrate that treating patellar fractures (PFs) using non-resorbable sutures as tension bands is a viable surgical option in terms of both clinical outcomes and complication rates at a mean follow-up of 5 years.

At the final follow-up, postoperative patient-reported outcome measures (PROMs) values showed improvement, consistent with the existing literature [4–7, 11], and no cases of healing defects were reported. However, five patients required secondary interventions. Two cases involved manipulation under anesthesia (MUA) due to knee stiffness and incomplete achievement of the desired range of motion (ROM), while three patients underwent suture removal due to superficial infection, skin irritation, and delayed wound healing.

Managing PFs presents a complex challenge for orthopedic surgeons, leading to the development of multiple surgical techniques and materials usage over the years [5–7, 9, 11]. Historically, metallic tension bands have been the gold standard technique [7], but recent studies have highlighted the high number of associated complications, prompting a reevaluation of their use [15–17]. Long-term outcomes studies have shown concerning rates of hardware removal attributed to factors such as subcutaneous hardware placement and wire knots causing skin irritation [15–17]. Due to the complications associated with metal implants, there has been increasing interest in nonabsorbable sutures, which have demonstrated superior failure load compared to metal implants in various orthopedic procedures [14, 18, 19].

The clinical results of this study align with similar studies comparing metallic and nonmetallic implants [9, 20–22], demonstrating good clinical outcomes with nonmetallic implants.

In the context of surgical procedures for patellar fracture reduction and synthesis, our findings reveal an operative efficiency, aligning closely with the benchmarks established by other researchers in the field [12, 14, 15, 23]. Notably, Shea et al. [23] have reported diminished surgical durations when employing non-resorbable sutures as tension bands compared to treatments involving metallic or hybrid methodologies. This observation underscores the potential advantages of utilizing non-resorbable sutures as a tension band technique, shedding light on a promising avenue for optimizing surgical procedures in patellar fracture management.

The main strength of this study lies in demonstrating the efficacy and good clinical results of nonabsorbable sutures as a tension band in PF treatment at mid-term follow-up, with a limited proportion of patients requiring further surgery, mainly due to minor hardware complications.

However, several limitations need to be considered, including the study's retrospective nature, single-center design, relatively short follow-up duration, lack of comparison between different implant types, and potential impact of external factors such as patient adherence to rehabilitation protocols and surgeon experience on outcomes.

## Conclusion

This study demonstrates that adopting nonmetallic fixation techniques with non-resorbable high-strength sutures is a reliable and promising alternative in the treatment of patellar fractures (PFs), potentially reducing complications and improving patient outcomes. However, further randomized clinical trials will be necessary to validate these results and ensure the broader applicability of this technique.

## Data Availability

Dataset analyzed in this study is available from the corresponding author on reasonable request.
